# Capsaicin-Induced Changes in LTP in the Lateral Amygdala Are Mediated by TRPV1

**DOI:** 10.1371/journal.pone.0016116

**Published:** 2011-01-13

**Authors:** Carsten Zschenderlein, Christine Gebhardt, Oliver von Bohlen und Halbach, Christoph Kulisch, Doris Albrecht

**Affiliations:** 1 Institute of Neurophysiology, Charité - Universitätsmedizin Berlin, CVK, Berlin, Germany; 2 Institute of Anatomy and Cell Biology, Ernst Moritz Arndt University, Greifswald, Germany; University of Cincinnati, United States of America

## Abstract

The transient receptor potential vanilloid type 1 (TRPV1) channel is a well recognized polymodal signal detector that is activated by painful stimuli such as capsaicin. Here, we show that TRPV1 is expressed in the lateral nucleus of the amygdala (LA). Despite the fact that the central amygdala displays the highest neuronal density, the highest density of TRPV1 labeled neurons was found within the nuclei of the basolateral complex of the amygdala. Capsaicin specifically changed the magnitude of long-term potentiation (LTP) in the LA in brain slices of mice depending on the anesthetic (ether, isoflurane) used before euthanasia. After ether anesthesia, capsaicin had a suppressive effect on LA-LTP both in patch clamp and in extracellular recordings. The capsaicin-induced reduction of LTP was completely blocked by the nitric oxide synthase (NOS) inhibitor L-NAME and was absent in neuronal NOS as well as in TRPV1 deficient mice. The specific antagonist of cannabinoid receptor type 1 (CB1), AM 251, was also able to reduce the inhibitory effect of capsaicin on LA-LTP, suggesting that stimulation of TRPV1 provokes the generation of anandamide in the brain which seems to inhibit NO synthesis. After isoflurane anesthesia before euthanasia capsaicin caused a TRPV1-mediated increase in the magnitude of LA-LTP. Therefore, our results also indicate that the appropriate choice of the anesthetics used is an important consideration when brain plasticity and the action of endovanilloids will be evaluated. In summary, our results demonstrate that TRPV1 may be involved in the amygdala control of learning mechanisms.

## Introduction

The transient receptor potential vanilloid type 1 (TRPV1) channel is a nonselective cation channel with high Ca^2+^ permeability that belongs to the TRP family of proteins [1]. TRPV1 was first identified due to its responsiveness to the pungent compound capsaicin. Capsaicin stimulates TRPV1 channels mainly located on polymodal C-fibers and initiates a complex cascade of events, including neuronal excitation, release of proinflammatory mediators, receptor desensitization and neurotoxicity [2]. TRPV1 is also activated by a wide range of stimuli including noxious heat (>42°C), protons, endogenous lipoxygenase products and fatty acid amides [3]. Mice lacking the TRPV1 gene demonstrate an impaired ability to develop inflammation-induced thermal hyperalgesia [4] and an increase in expression of calcitonin gene-related peptide [5].

In male mice, TRPV1 receptors have been mapped to the prefrontal cortex, nucleus accumbens, amygdala, and hippocampus. The amygdala has been shown to exhibit a high degree of plasticity in various models of long-term synaptic modification, including long-term potentiation (LTP). Pain has a strong emotional component and persistent pain is significantly associated with depression and anxiety disorders. Whereas a key role of the central nucleus of the amygdala (CE) has been established in integration of nociceptive information, the concept of the lateral nucleus of the amygdala (LA) as an important contributor to pain and its emotional component is still emerging. A recent report indicates that TRPV1 receptors are involved in promoting unconditioned and conditioned fear [6]. Following auditory fear conditioning, TRPV1^−/−^ mice also showed less freezing to the tone and conditioning context. These impairments were accompanied by reduced hippocampal LTP. Fear conditioning is amygdala dependent [7]. The LA receives direct sensory inputs from the thalamus and cortex, serving as the sensory input station of the amygdala [8]. The LA sends direct and indirect projections to the CE, which in turn projects to the brainstem and the hypothalamic regions that govern defensive behaviors and accompany autonomic and endocrine responses [8]. Several studies indicate that both fear conditioning-induced neuronal plasticity and LTP at amygdaloid synapses share common mechanisms of induction and expression [9], [10]. The phenomenon of LTP, a lasting increase in synaptic efficacy following brief, intense activation of afferences terminating on synapses in the LA has been studied nearly exclusively in coronal brain slices. In coronal slices, synaptic responses were either elicited by stimulation of fibers from the thalamus [11]–[14] or the external capsule (EC). The EC contains amygdala afferences from higher-order sensory cortices [15]. In horizontal slices, EC stimulation also activates excitatory afferences from cortical structures and includes afferences from the lateral entorhinal and perirhinal cortex that course through the EC and synapse in the lateral and the basolateral nucleus of the amygdala [16]. Stimulation within the LA also activates local connections within the LA and afferences from other amygdaloid nuclei [16]. The amygdala lacks an elongated structural organization compared to other brain regions [17]–[20] and is therefore not subject to anisotropic conductance [21], [22]. As a result, the field potential response in the LA is not solely dependent on underlying dendrite alignment, allowing synaptic activity to potentially contribute to the response [23]. Field potential recordings have been extensively performed in the LA to study monosynaptic plasticity not only in the behaving animal, but also in brain slices [24], [25]. Studies performed in coronal brain slices have shown that high frequency stimulation (HFS) [26] is able to induced persistent LA-LTP only if the postsynaptic cells were additionally depolarized [14] or GABA receptor antagonists were applied [27], [28]. In contrast, LA-LTP in horizontal brain slices can be induced either by HFS of EC-afferents [29], [30] or of intranuclear fibers (IN-stimulation) [30], [31] without inhibiting GABA receptors. Whereas basal transmission in the LA in horizontal slices is dependent on AMPA and kainate receptors [32]–[34], we could demonstrate that LTP induction in horizontal slices depends on NMDA receptors for both used inputs [32]. In addition, EC-induced LTP in horizontal slices is also dependent on L-type voltage-gated calcium channels [32]. Importantly, we also could induce stable LA-LTP by stimulation of intranuclear afferents in recordings with sharp microelectrodes [34] without adding GABA receptor antagonists and without additional depolarization of the postsynaptic cell.

Here we show that TRPV1 proteins are expressed in neuronal and non-neuronal cells of the LA. Capsaicin did not change spontaneous miniature excitatory postsynaptic currents (mEPSCs) or miniature inhibitory postsynaptic currents (mIPSCs) recorded in LA projection neurons. The main aim of this study was to analyze capsaicin-induced changes in the magnitude of LTP in the LA (LA-LTP). Since the mechanisms in induction of LA-LTP are dependent on the used afferents we have attempted to determine the capsaicin-induced effect on LA-LTP depending on the used input (EC stimulation versus intranuclear stimulation site (IN)) in horizontal slices. To compare our data with these from Li et al. [35] we also examined whether our findings can be reproduced in coronal slices derived from juvenile and adult animals. A further aim was to investigate whether capsaicin-induced effects in the amygdala primarily involve endogenous nitric oxide synthase (NOS) responsible for the synthesis of nitric oxide (NO). Moreover, we addressed whether the suppressive effect of capsaicin on LA-LTP might be mediated via CB1 receptors, and to what extent, sensitization of TRPV1 can influence the capsaicin-induced effect on LA-LTP.

## Results

### TRPV1 proteins are expressed in the LA

**Figure 1:**
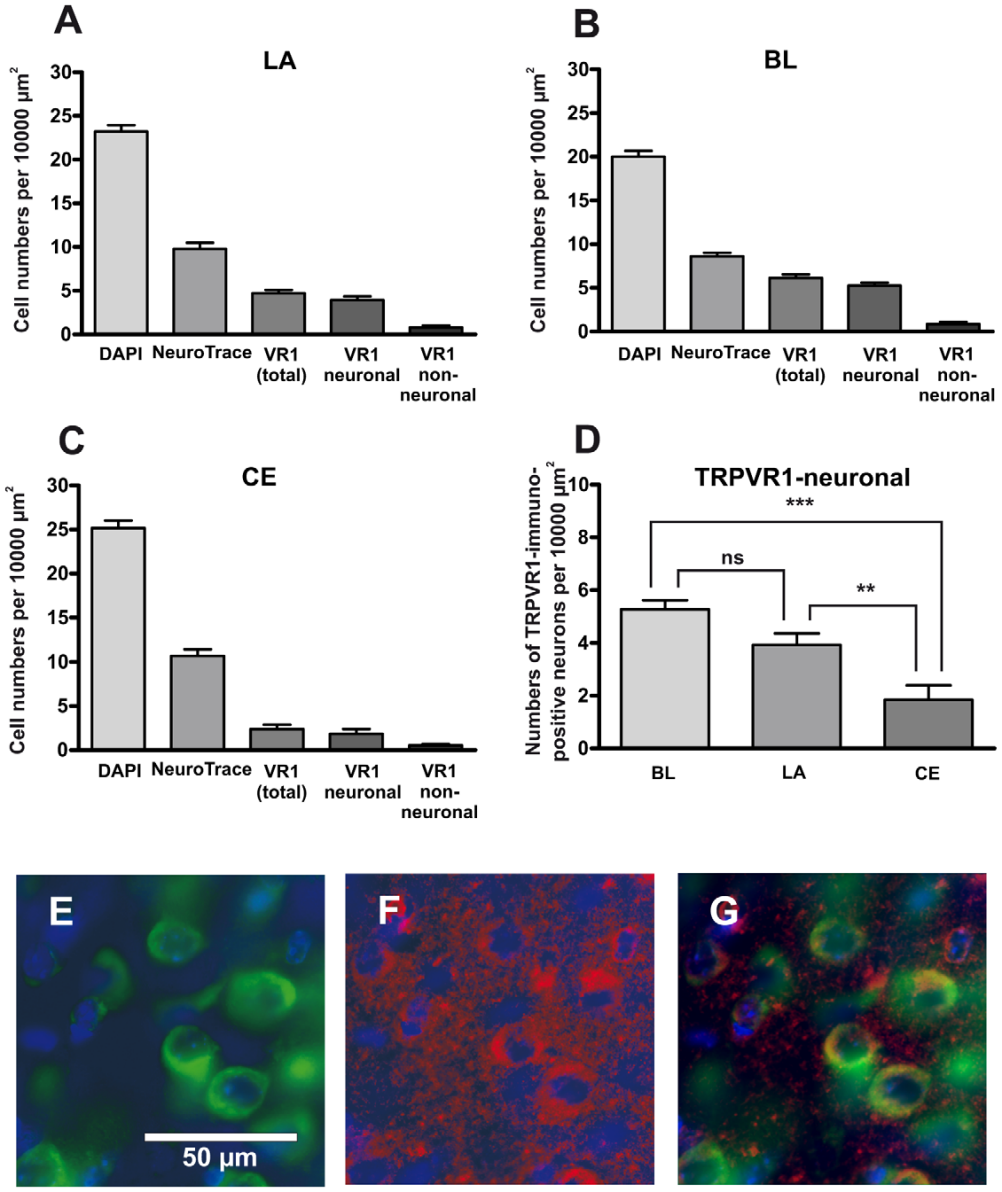
TRPV1 protein expression within the amygdala. (A–C) These graphs illustrate the densities of cell nuclei (visualized with DAPI), neurons (visualized with NeuroTrace), cells expressing TRPV1 (visualized by immunohistochemistry) as well as the densities of neuronal and non-neuronal cells expressing TRPV1 in the lateral nucleus (A), basolateral (B) and central nucleus (C) of the amygdala. (D) The numerical neuronal density of TRPV1 immunopositive neurons in the basolateral amygdala (LA, BL) is significantly higher than in the CE, as determined by ANOVA followed by a Tukey's posthoc test (ns: non-significant; **: p≤0.01; ***: p≤0.001). (E–G) Example of a triple-stained section (DAPI, NeuroTrace and anti-TRPV1; lateral nucleus of the amygdala). NeuroTrace was used to visualize neuronal cells (in green, E). In the same section, binding of TRPV1 antibodies was visualized with Cy5 (in red, F). Figure G displays the merged images of E and F (in each case DAPI (in blue) was used to visualize cell nuclei).

The highest level of TRPV1 immunoreactivity was detected in the hippocampus and cortex, as previously reported [36], [37]. Moreover, TRPV1 immunoreactivity was also detected in the amygdala, albeit at a lower level. Concerning the amygdala, the number of cells within a Region of Interest, ROI (10.000 µm^2^) was determined as well as the number of neurons, the total number of TRPV1 expressing cells, the number of TRPV1-immunopositive neuronal and non-neuronal cells (the results are summarized in Fig. 1). Analysis of the lateral (LA), basolateral (BL) and central nucleus (CE) of the amygdala revealed that TRPV1 protein mainly localizes to neuronal cells (Fig. 1A–C). The mean numbers of neurons per ROI (“neuronal density”) were 10.69±0.73 (CE), 9.78±0.70 (LA) and 8.60±0.41 (BL). Despite the fact that the CE displays the highest neuronal density, the highest number of TRPV1 labeled neurons per ROI (“density”) was found within the nuclei of the basolateral complex of the amygdala (BL: 5.27±0.35; LA: 3.93±0.44; CE: 1.85±0.55). ANOVA followed by a Tukey's posthoc test revealed that the numerical densities of TRPV1 immunopositive neurons in the basolateral complex (LA as well as BL) were significantly higher than in the CE (Fig. 1D).

### Capsaicin dose-dependently reduces LA-LTP in horizontal slices

**Figure 2:**
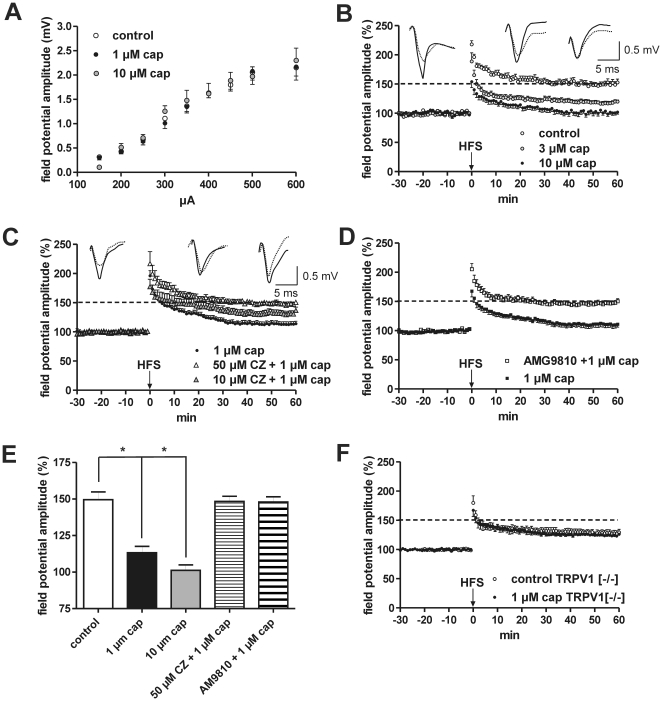
The capsaicin-induced reduction of LTP recorded in horizontal slices is blocked by TRPV1 antagonists and is absent in TRPV1 deficient mice. (A) Input-output curves as evoked by single stimuli applied at EC fibers. Drug-free control recordings (n = 15) did not differ from recordings made in capsaicin-treated slices (1 µM cap: n = 15; 10 µM cap: n = 8). (B) EC stimulation caused a stable LA-LTP in horizontal slices derived from adult male mice. Bath-applied capsaicin (cap) induced a dose-dependent reduction in the magnitude of LA-LTP. Data points represent averaged amplitudes (mean ± SEM) normalized with respect to baseline values. Representative traces were recorded 5 min prior to tetanus (dashed lines) and 60 min after tetanus (solid lines). (C) Capsazepine completely blocks the capsaicin-induced reduction of LTP only at a concentration of 50 µM. (D) The capsaicin-induced reduction of LA-LTP could be also blocked by the specific TRPV1 antagonist AMG9810. (E) Bar histogram of data points averaged 57 to 60 min after HFS and normalized with respect to baseline (mean ± SEM). Significant differences are indicated. *p<0.05. (F) In TRPV1^−/−^ mice the inhibitory effect of 1 µM capsaicin was absent. HFS: high-frequency stimulation.

Although our results show that TRPV1 proteins are expressed by LA neurons, their specific function and role is yet to be established. Using capsaicin as a pharmacological activator of TRPV1 we first analyzed the input/output curves of extracellular recordings. We found that neither 1 µM nor 10 µM capsaicin altered basal transmission (Fig. 2A). In addition, capsaicin did not change the extracellularly recorded action potential frequency in amygdala slices which were perfused with “zero-Mg^2+^” artificial cerebrospinal fluid (ACSF) (data not shown). However, we found that it dose-dependently (cap: 0.1–10 µM) reduced the magnitude of LA-LTP induced by HFS of external capsule (EC) fibers (control: 149.6 ± 5,3% [n = 13] vs. 0.1 µM cap: 133.1 ± 7.0% [n = 8] vs. 3 µM cap: 118.8 ± 2.9% [n = 9] vs. 10 µM cap: 101.2 ± 3.7% [n = 8], p<0.05; Fig. 2B), indicating that TRPV1 receptors may play a role in the synaptic plasticity of the amygdala. A similar suppressive effect of 1 µM capsaicin on HFS-induced LA-LTP was obtained when afferents within the LA were stimulated both in males and females (males: 142.6 ± 5.1% [n = 9] vs. 118.7 ± 6.9% [n = 9]; females: 140.8 ± 8.5% [n = 8] vs. 120.1 ± 6.5% [n = 7], p<0.05; data not shown). Thus, in contrast to the data from the hippocampus [35], capsaicin reduced the magnitude of LA-LTP in horizontal slices. To examine whether capsaicin-induced suppression of LTP is mediated by TRPV1 receptors, we analyzed the effects of capsaicin in the presence of capsazepine (CZ), a TRPV1 receptor antagonist. A concentration of 10 µM CZ partially blocked the capsaicin-induced effects on LA-LTP. In the presence of 50 µM capsazepine, capsaicin failed to induce any inhibitory effects on LTP (1 µM cap: 113.3 ± 4.3% [n = 14] vs. 10 µM CZ +1 µM cap: 132.4 ± 5.0% [n = 8] vs. 50 µM CZ +1 µM cap: 148.4 ± 3.4% [n = 12], Fig. 2C, E). Due to the species-specific action of capsazepine, a high concentration of the TRPV1 antagonist [38] may be required. The capsaicin-induced reduction of LA-LTP could be also blocked by the specific TRPV1 antagonist AMG9810 (1 µM cap: 110.4±5.5% [n = 12] vs. 1 µM AMG9810 +1 µM cap: 147.9±3.6% [n = 7]; Fig. 2D, E). These results support the conclusion that the capsaicin-induced reduction of LA-LTP is mediated by TRPV1 receptors. In addition, we show that the capsaicin-induced suppression of EC-induced LA-LTP is absent in TRPV1^−/−^ mice (control: 127.8 ± 4.9% [n = 11] vs. 124.2 ± 1.9% [n = 9]; Fig. 2F).

### GABA transmission is not involved in mediating capsaicin-induced effects on LA-LTP

**Figure 3:**
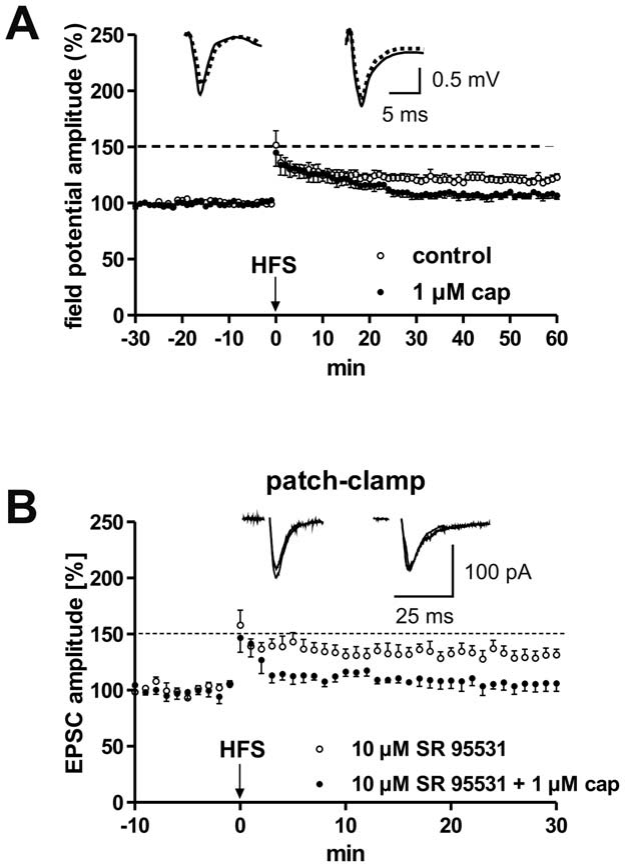
EC-induced LTP is also suppressed by capsaicin in coronal brain slices. This suppression cannot be blocked in the presence of 10 µM SR95531. (A) In extracellular recordings the magnitude of LA-LTP obtained in coronal slices derived from juvenile mice is lower than that in horizontal slices. Nevertheless, capsaicin reduced LA-LTP in both instances. (B) In patch clamp recordings the complete blockade of GABA_A_ receptors did not change the inhibitory effect of capsaicin on LA-LTP obtained in coronal slices derived from adult mice. Representative traces were recorded 5 min prior to tetanus (dashed lines) and 60 min and 30 min, respectively, after tetanus (solid lines) in the lateral nucleus of the amygdala.

It can be hypothesized that capsaicin-induced reduction of LA-LTP might be due to an increase in the release of inhibitory neurotransmitters such as GABA, because TRPV1 activation can lead to the enhancement of inhibitory neurotransmission, at least in a small portion of substantia gelatinosa neurons [39]. TRPV1 activation was shown to potently enhance glutamate release onto GABAergic terminals, facilitating GABA release in the dorsal vagal complex [40]. Additionally, the stimulation of TRPV1 channels was found to mediate long-term depression at synapses on hippocampal interneurons [41]. In order to investigate the possible involvement of GABAergic interneurons in the inhibitory effect of capsaicin on LA-LTP, we co-administered the selective GABA_A_ receptor antagonist SR95531 (SR) and capsaicin. To avoid a desynchronization of LA neurons and epileptic seizures, we used low concentrations of SR in extracellular recordings. The effects of SR at a concentration of 0.1 µM suggest an additive action of both substances (SR: 164.6±5.7% [n = 10] vs. SR +1 µM cap: 130.6±3.9% [n = 10]; p<0.05). Next, the concentrations of Mg^2+^ and of Ca^2+^ in the ACSF were increased to 3 mM to reduce the excitability in the brain slices. Under these conditions, HFS was unable to evoke LA-LTP. In slices that were pretreated with 10 µM SR, an enhancement of field potential amplitudes after HFS was observed; however, capsaicin remained inhibitory (SR: 128.2±5.6% [n = 10] vs. SR +1 µM cap: 105.1±3.5% [n = 10], p<0.05, data not shown). Further recordings made in high Mg^2+^ and Ca^2+^ conditions, showed that blockade of GABA_B_ receptors with 1 µM CGP55845 (CGP) was also unable to block the capsaicin-induced inhibition of LA-LTP (CGP: 144.4±7.8% [n = 5] vs. CGP +1 µM cap: 114.6±4.3% [n = 9], p<0.05, data not shown). To verify this data, we performed patch clamp recordings. In these LTP experiments we stimulated EC fibers in coronal slices. In horizontal slices, EC stimulation activates excitatory afferences from cortical structures, including afferences from the lateral entorhinal and perirhinal cortex that course through the EC and synapse in the LA and the BL [16]. In contrast, in coronal slices the EC contains amygdala afferences from higher-order sensory cortices [15]. It has been shown that inhibitory mechanisms in horizontal slices are weaker than in coronal slices [42]. In accordance with these investigations we directly show for the first time that the magnitude of LA-LTP is weaker in coronal than in horizontal slices. However, the suppressive effect of capsaicin on LA-LTP was also present in coronal slices derived from juvenile mice (control: 120.5±3.2% [n = 8] vs. 1 µM cap: 107.3±3.5% [n = 7], p<0.05, Fig. 3A). To get a complete blockade of GABAergic transmission in coronal slices derived from adult mice, we tested the effects of co-administered SR (10 µM) and capsaicin (1 µM) on LTP in patch clamp recordings of EPSCs. This co-application also did not block the suppressive action of capsaicin on LA-LTP (SR: 130.2±6.0% [n = 5] vs. SR +1 µM cap: 106.0±6.9% [n = 4], Fig. 3B).

**Figure 4:**
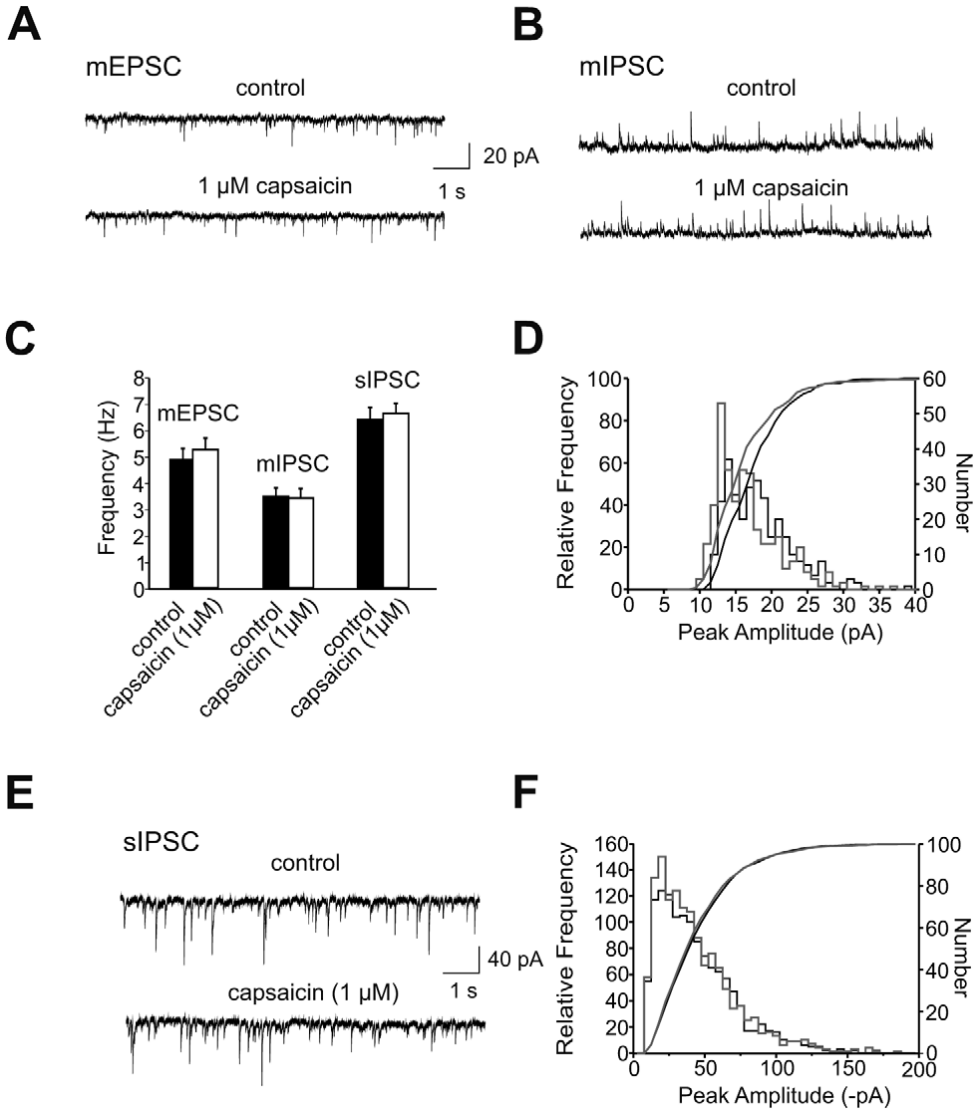
Properties of spontaneous PSCs in the absence and presence of TTX. (A) Continuous recording shows spontaneous EPSCs in the presence of TTX (mEPSCs) from a LA projection neuron under control conditions and 10 min after bath application of capsaicin (1 µM), V_m_ = −70 mV. (B) Continuous recording shows spontaneous IPSCs in the presence of TTX (mIPSCs) from another LA projection neuron under control conditions and 10 min after bath application of capsaicin (1 µM), V_m_ = 0 mV. (C) Summary data shows mean frequency of mEPSCs, mIPSCs and sIPSCs under control conditions and in the presence of capsaicin, SEM is indicated by error bars. (D) Amplitude distributions of mIPSCs recorded in under control conditions (black) and in the presence of 1 µM capsaicin (grey). Superimposed lines show cumulative amplitude histograms (p>0.05, Kolmogoroff-Smirnov test). (E) Continuous recording shows spontaneous IPSCs in the absence of TTX (sIPSCs) under control conditions and 10 min after bath application of capsaicin (1 µM), V_m_ = −70 mV. (F) Amplitude distributions of sIPSCs recorded in under control conditions (black) and in the presence of 1 µM capsaicin (grey). Superimposed lines show cumulative amplitude histograms.

To assess whether capsaicin affects spontaneous inhibitory network activity, spontaneous inhibitory postsynaptic currents (sIPSCs) were recorded at a holding potential of -70 mV using a CsCl based internal solution. sIPSCs were pharmacologically isolated by application of CNQX (20 µM) and APV (30 µM). As shown in Fig. 4E and F, bath application of capsaicin (5 µM) did not alter the frequency of sIPSCs and had no effect on the amplitude distribution [n = 6].

Thus, and corresponding to the results obtained in horizontal brain slices, the suppressive effect of capsaicin on LA-LTP does not seem to be due to an activation of GABAergic transmission.

### Capsaicin neither affected frequency nor amplitude of mEPSCs or mIPSCs

To determine whether the capsaicin effects might be due to changes in presynaptic release probability, we recorded miniature excitatory and inhibitory postsynaptic currents (mEPSCs and mIPSCs) from LA projection neurons of juvenile mice (P18–28) in the presence of the sodium channel blocker tetrodotoxin (TTX, 1 µM) using a Cs-gluconate based internal solution. mEPSCs were recorded at a holding potential of −70 mV in the presence of GABA receptor antagonist bicuculline (5 µM) and mIPSCs were recorded at a holding potential of 0 mV in the presence of glutamate receptor blockers CNQX (20 µM) and APV (30 µM). Fig. 4A shows representative traces under control conditions (upper trace) and 10 min after bath application of 1 µM capsaicin (lower trace). Recordings of mIPSCs from a different cell (upper trace) and after application of 1 µM capsaicin (lower trace) are shown in Fig. 4B. Bath application of 1 µM capsaicin changed neither the frequency (Fig. 4C) nor the amplitude of mEPSCs [n = 6] and mIPSCs [n = 6] (Fig. 4D). These data indicate that capsaicin does not increase glutamate or GABA release from presynaptic terminals in LA neurons under basal transmission conditions.

### NO and CB1 receptors are involved in mediating capsaicin-induced reduction of LA-LTP

**Figure 5:**
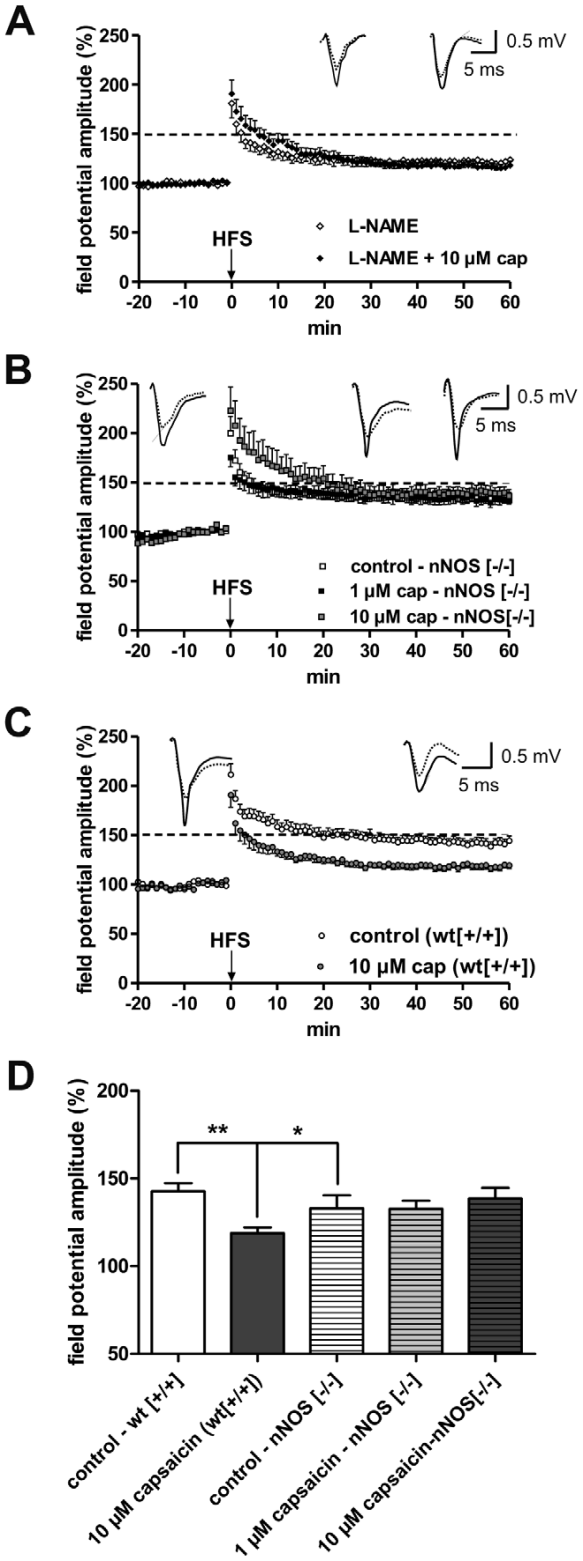
NO is involved in the mediation of the suppressive effect of capsaicin on LA-LTP. (A) The application of the unspecific NOS antagonist L-NAME caused a reduction of LA-LTP and blocked the capsaicin-induced suppression of LA-LTP. (B) In nNOS deficient mice the suppressive effect of capsaicin on LA-LTP is again absent. (C) In wild-type mice the decrease in LA-LTP induced by capsaicin was reproduced. Representative traces were recorded 5 min prior to tetanus (dashed lines) and 60 min after tetanus (solid lines) in the LA. (D) Bar histogram of data points averaged 57 to 60 min after HFS and normalized with respect to baseline (mean ± SEM). Significant differences are indicated. *p<0.05. Recordings were done in horizontal slices derived from adult mice.

For the medial amygdala it has been shown that capsaicin significantly increases the expression of neuronal NOS (nNOS) mRNA and protein, as demonstrated by in-situ hybridization and immunohistochemistry [43]. Based on that, we investigated whether changes in NO production are responsible for the suppressive effects of capsaicin on LA-LTP. As shown in Fig. 5A, pretreatment with L-NAME (200 µM), an unspecific NOS inhibitor, blocked the reduction of capsaicin-induced LA-LTP (L-NAME: 121.3±5.9% [n = 8] vs. L-NAME +1 µM cap: 117.5±3.5% [n = 12], p>0.05). L-NAME *per se* reduced the amplitude of field potentials after HFS, implicating a role of NO in LA-LTP, as demonstrated previously in coronal slices [44]. Moreover, the capsaicin-induced effect was absent in nNOS deficient mice (control: 133.0±7.4% [n = 10] vs. 1 µM cap: 132.7±4.6% [n = 6] vs. 10 µM cap: 138.6±6.1% [n = 7], p>0.05; Fig. 5B), but was reproduced in wild-type mice (control: 142.7±4.6% [n = 8] vs. 10 µM cap: 118.8±3.3% [n = 8], p<0.05; Fig. 5C,D).

**Figure 6:**
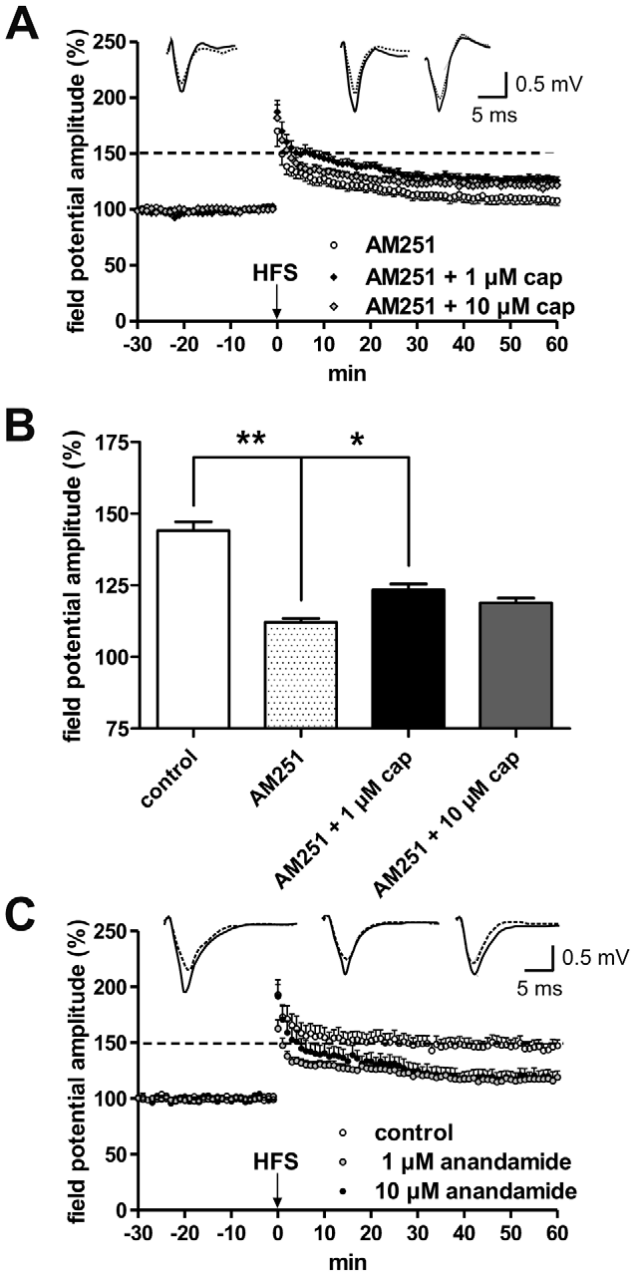
The CB1 receptor is involved in the mediation of capsaicin-induced inhibition of LA-LTP. (A) The CB1 receptor antagonist AM251 (2.5 µM) evoked a reduction of LA-LTP in comparison with controls. This reduction was not enhanced by capsaicin. In contrast, the co-administration of AM251 and capsaicin (1, 10 µM) caused an increase in the magnitude of HFS-induced LA-LTP. (B) Bar histogram of data points averaged 57 to 60 min after HFS and normalized with respect to baseline (mean ± SEM). Significant differences are indicated. *p<0.05, **p<0.01. (C) Anandamide (1 µM, 10 µM) provoked a significant suppression of LA-LTP. Representative traces were recorded 5 min prior to tetanus (dashed lines) and 60 min after tetanus (solid lines). Data were obtained by using horizontal slices derived from adult mice.

Outside the brain anandamide significantly decreases NOS activity via CB1 receptors, whereas TRPV1 stimulation by anandamide enhances NOS activity [45]. To get insight into a comparable mechanism within the brain, we used the CB1 receptor antagonist AM251 (2.5 µM) for further experiments in horizontal slices. As shown in Fig. 6A and B, AM251 suppressed HFS-induced LA-LTP. However, co-application of AM251 and capsaicin reduced the inhibitory effect of capsaicin on the magnitude of LA-LTP (AM251: 108.8±4.3% [n = 9] vs. AM251 +1 µM cap: 126.8±2.8% [n = 10] vs. AM251+10 µM cap: 120.9±3.5% [n = 8], p<0.05). This suggests that the suppressive effect of capsaicin on LA-LTP might be mediated by the synthesis of anandamide or other endocannabinoids and the activation of CB1 receptors. Furthermore, application of anandamide (1 µM: 117.5±2.2% [n = 11], 10 µM: 110.4±5.5% [n = 12]) caused the same inhibitory effect as capsaicin on LA-LTP (Fig. 6C). We found similar results with N-oleoyldopamine (OLDA) (data not shown), a bioactive lipid originally found in the mammalian brain. This endovanilloid selectively activates the TRPV1 channel.

### Sensitization of TRPV1 enhances LA-LTP

**Figure 7:**
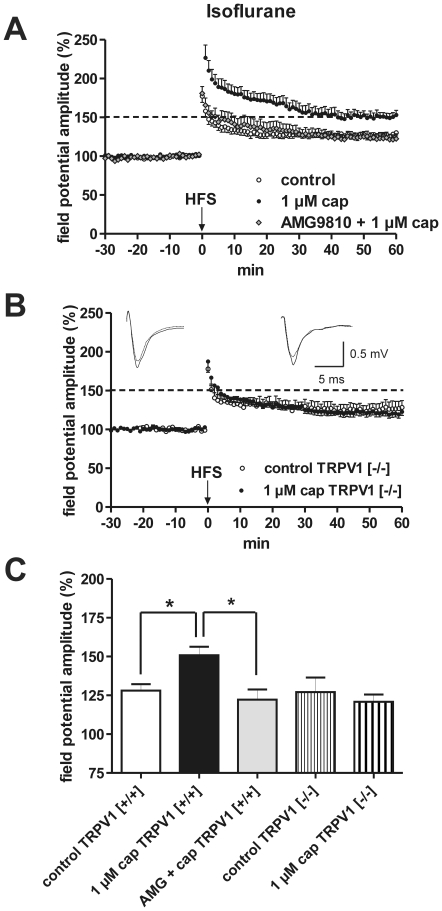
Isoflurane anesthesia before euthanasia instead of ether caused a capsaicin-induced enhancement of LA-LTP in horizontal slices derived from adult mice. (A) HFS-induced LTP is increased in magnitude by 1 µM capsaicin in comparison with control. This increase can be blocked by the specific TRPV1 antagonist AMG9810. (B) Capsaicin-induced LTP enhancement is absent in TRPV1^−/−^ mice. Representative traces were recorded 5 min prior to tetanus (dashed lines) and 60 min after tetanus (solid lines). (C) Bar histogram of data points averaged 57 to 60 min after HFS and normalized with respect to baseline (mean ± SEM). Significant differences are indicated. *p≤0.05.

The standard inhalant anesthetic for experiments in brain slices is isoflurane and it replaced the flammable ethers used in the pioneer days of surgery. As it recently has been shown that isoflurane causes a sensitization of TRPV1 [46]–[48], we compared the effect of capsaicin in mice euthanized after deep isoflurane anesthesia with that of ether anesthesia. As shown in Fig. 7A, capsaicin caused a significant enhancement of the magnitude of EC-induced LA-LTP in the isoflurane group (control: 128.1±4.1% [n = 10] vs. 1 µM cap: 150.8±5.4% [n = 8]; p<0.05) as described for the hippocampus [35]. This enhancement could be blocked by the TRPV1 antagonist AMG9810 (1 µM AMG9810 +1 µm cap: 122.3±6.5% [n = 8]; Fig. 7A). In TRPV1^−/−^ mice, the capsaicin induced effect on LTP was missing (Fig. 7B). It is to note that decapitation after isoflurane anesthesia provokes a reduced magnitude of LTP as compared to that after ether anesthesia (Fig. 7A, C).

## Discussion

Our immunohistochemical results support previous results that TRPV1 is expressed in the amygdala [49]. The expression of TRPV1 in the LA is higher than in the CE which is defined as the “nociceptive amygdale” [50]. The functional role of TRPV1 receptors located in the LA is not clear, since these cells are unlikely to be exposed to capsaicin or noxious heat directly. However, TRPV1^−/−^ mice have reduced anxiety and diminished fear conditioning [6].

For the first time, it was demonstrated that capsaicin caused a dose-dependent decrease in HFS-induced LA-LTP. The suppressive effect of capsaicin was not age-dependent and did not show input-specificity. Our results further show that capsaicin-induced suppression of HFS-induced LA-LTP is mediated by the stimulation of TRPV1 receptors, since capsazepine and AMG9810 blocked the capsaicin-induced effect and the effect was absent in TRPV1 deficient mice. The experiments with GABA_A_ receptor antagonists led us assume that GABAergic interneurons are not involved in the mechanisms of capsaicin-induced plastic changes. Although it has been shown that presynaptic GABA_B_ receptors suppress LA-LTP induced by EC stimulation, they only do so in projection neurons and not in GABAergic interneurons [51]. Our results suggest that GABA_B_ receptors are not involved in mediating capsaicin-induced suppression of LA-LTP.

We did not find capsaicin-induced changes in presynaptic neurotransmitter release in the LA as compared to data obtained in other brain structures [52], [53]. Our patch clamp recordings are in agreement with the unaltered input/output curves after capsaicin application.

**Figure 8:**
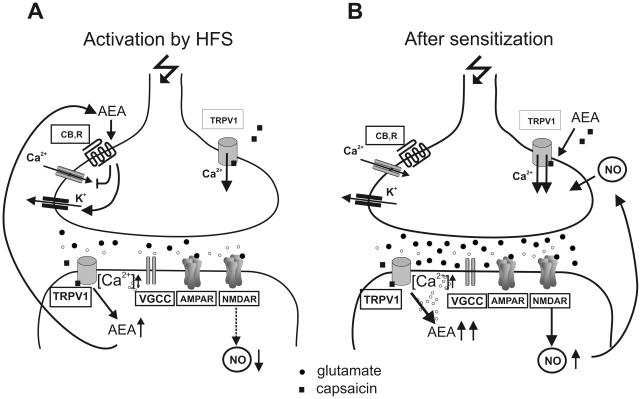
Amygdala vanilloid versus cannabinoid receptors. (A) Schematic representation of potential TRPV1-CB1 crosstalk in the amygdala after HFS (ether anesthesia). Capsaicin induces postsynaptically the synthesis of anandamide (AEA). Glutamate release is reduced through the action of AEA at CB1 presynaptically. The lower activation of NMDARs results in a diminished NOS activation, leading to a reduction of LA-LTP. (B) After sensitization of TRPV1 (isoflurane anesthesia) levels of AEA are elevated and stimulate presynaptic TRPV1 besides capsaicin. An increase in glutamate release stimulates the NO synthesis which presynaptically enhances release of glutamate. This resulted in an enhancement of LA-LTP.

Earlier data have shown that capsazepine can prevent cannabinoid-mediated inhibition of EPSCs in CA1 pyramidal cells [54], thus, interplay of TRPV1 channels with CB1 receptors can be expected. Our results strongly support the assumption that capsaicin specifically modulates the NO system. L-NAME pretreatment clearly reduced the magnitude of LA-LTP; however, the co-application of L-NAME and capsaicin blocked the capsaicin-induced reduction of LA-LTP. The specific modulation by capsaicin may be valid for both the endothelial NOS and the nNOS. Our results show that at the least nNOS is involved in mediating the capsaicin-induced effect. It should be noted that both L-NAME and the nNOS deficiency in knockout mice *per se* reduced the magnitude of LA-LTP. Similar effects have been described in other investigations [44]. Our observations indicate that modulation of the endogenous NOS system and production of NO constitutes a major pathway through which capsaicin acts in the amygdala (see also Fig. 8).

Additionally, the present study strongly supports the idea that endogenous vanilloids can cause pain modulation mediated directly by TRPV1 receptors. Anandamide, an endocannabinoid acting predominantly on CB1 receptors, has been implicated as an agonist at the TRPV1 receptor [55], [56]. Anandamide is able to bind to TRPV1 proteins at higher concentrations [57]. Experiments with CB1 blockade and experiments done in nNOS deficient mice led us to assume that postsynaptic TRPV1 activation results in the release of cannabinoids, and consequently in the suppression of NOS as shown in the periphery [58] via NMDARs (Fig. 8). It has been shown that TRPV1 activation regulates anandamide synthesis and anandamide metabolites affect TRPV1 responses [59].

Our findings suggest a retrograde endocannabinoid signalling followed by the release of anandamide. A presynaptic CB1 stimulation via anandamide will result in a reduction of glutamate release and therefore in a reduced activation of NMDARs. It is well known that Ca^2+^ influx through NMDAR triggers the synthesis of NO by activating the enzyme nNOS in postsynaptic densities. It is important to note that fatty acid amide hydrolase (FAAH) catalyzes the hydrolysis of the endogenous cannabinoid anandamide. FAAH has been shown to be (among others) strongly expressed in the basolateral complex of the amygdala (BLA; composed of the LA, BL and the basomedial nucleus), but is nearly absent in the central complex of the amygdala of rats [60]. This indicates that anandamide can be synthesized within the BLA. It is widely recognized that anandamide is not stored in vesicles like other mediators but by analogy with other eicosanoids, it is produced ‘on demand’ in a Ca^2+^-dependent manner. This release of cannabinoids seems to be responsible for the capsaicin-evoked suppression of LA-LTP.

In accordance with results obtained in the hippocampus [61], AM251 inhibited the magnitude of LA-LTP *per se*. The AM251-induced LTP suppression in the hippocampus was interpreted by the action of AM251 over GABAergic interneurons that (indirectly) modulate the glutamatergic, LTP-bearing pyramidal cells. Furthermore, it is known that AM251 might be acting as an inverse agonist at CB1 receptors. This can explain the inhibitory effect of AM251 on the magnitude of LA-LTP. Cannabinoids can reduce both excitatory and inhibitory synaptic transmission via CB1 receptors in the LA [62]. Since we could not detect an involvement of GABAergic interneurons in the capsaicin-induced effect on LA-LTP, the release of glutamate seems to be reduced by CB1 activation (Fig. 8). It can be postulated that cannabinoid receptor activation does not directly inhibit the molecular mechanisms responsible for long-term synaptic plasticity, but instead impairs LTP by reducing presynaptic glutamate release. As a result, the postsynaptic membrane is not sufficiently depolarized to relieve Mg^2+^ blockade of NMDA receptors completely.

Thus, our results support the concept that anandamide modulates NO levels by two independent pathways: (1) diminishing the NOS activity via cannabinoids (ether group); and (2) stimulating NO synthesis via TRPV1 (isoflurane group). In addition, it has been shown that anandamide inhibits the metabolism and physiological actions of 2-arachidonoylglycerol. The interaction between these both endocannabinoids was lost after pharmacological or genetic inactivation of TRPV1 channels [63]. TRPV1-mediated synaptic depression rather than potentiation has also been reported at synapses of the dentate gyrus, supporting such a mechanism in central neurons [64].

Our results also indicate that the method of anesthesia is an important consideration when brain plasticity and the action of endovanilloids will be evaluated. We also found that capsaicin evokes an enhancement of LA-LTP in ethanol-treated slices (10 mM, unpublished data). It is known that ethanol is also able to sensitize TRPV1 [65]. Our results demonstrated that a short deep isoflurane anesthesia can influence brain plasticity for hours at least if recordings were performed in an interface chamber. It is known that submerged chambers offer significant experimental advantages, including fast exchange of pharmacological agents and visually guided patch-clamp recordings. However, a major advantage of the interface chamber is that fluid shunting is minimized which increases the size of the recorded extracellular field potential. Previous studies on amygdala functions were commonly performed in coronal slices when GABAergic transmission was inhibited. In these conditions the effect of isoflurane which mainly affect GABA_A_ receptors [66]–[68] might not be apparent.

It can be suggested that sensitization of TRPV1 by endovanilloids might induce a strong impact on brain plasticity in the amygdala due to an overexcitation. It has been shown that chronic psychoemotional stress impairs cannabinoid-receptor-mediated control of GABA transmission at least in the striatum [69]. In the range of the normal behavior the synthesis of endocannabinoids acting at the CB1 receptor could limit this overexcitation.

Currently, we only can speculate how TRPV1 receptors in afferents to the LA may be activated besides endocannabinoids. Pathological changes in brain temperature or pH, for example after a severe stroke [70], may influence TRPV1 activity, but normal brain pH and temperature are unlikely to result in TRPV1 activation *per se*. Given an important role of TRPV1 in neuronal plasticity, as it can be suggested based on our data and data from others [6], [35], systemic administration of TRPV1 antagonists may interfere with plastic changes attributed to learning and memory. Thus, drugs targeting TRPV1 may adversely affect cognitive function, representing a potential roadblock to the usage of TRPV1 antagonists to treat pain [71].

## Methods

### Animals

For immunohistochemistry, adult C57BL/6 mice were used. For electrophysiological experiments, juvenile (18–23 days) and adult male C57BL/6 mice (8–12 wk) were used. Age-matched TRPV1 knockout mice (B6.129S4-Trpv1^tm1Jul^/J) as well as breeder pairs for B6,129S-Nos1^tm1Plh^ (nNOS^-/-^) and C57BL/6J (wt) were obtained from The Jackson Laboratory (Maine, USA). Heterozygous (+/−) mice were breed to produce nNOS^-/-^ mice. Homozygous (+/+) mice of this breeding served as controls. At the time of electrophysiological recordings wt and nNOS^−/−^ mice were 12–14 months old. Breeding was continuously monitored by assessing the genetic status of the animals via polymerase chain reaction. Genetic testing using the tip of the mouse-tail was done according to the protocols of The Jackson Laboratory (Maine, USA). Animals were housed in standardized conditions with an artificial 12-h dark-light cycle and a room temperature of 22°C. Mice had free access to food and water. All of the experimental protocols were approved by government authorities (T0344/05) and conformed to the European Communities Council Directive of November 24, 1986 (86/609/EEC). All efforts were made to minimize suffering.

### Immunohistochemistry

Adult C57BL/6 mice (n = 3) were euthanized with ether. Afterwards, they were transcardially perfused with phosphate buffered saline followed by perfusion with 4% paraformaldehyde. The brains were removed and postfixed in 4% paraformaldehyde for 24 h at 4°C. 25 µm thick serial coronal sections were made using a vibratom (Leica VT1000, Germany) and mounted onto gelatin-coated slides. Sections were prepared for triple-staining (anti-TRPV1 (1), NeuroTrace (2) and DAPI (3)).

(1) Sections were incubated in a solution containing 1% normal goat serum, 0.3% Triton-X100 and the primary antibody (ab 31895: anti-TRPV1 1:1000; abcam, USA) over night at 4°C. The primary rabbit polyclonal antibody is directed against a KLH-conjugated peptide that corresponds to the C-terminal amino acids 825-839 of the murine TRPV1. After rinsing the sections were transferred to biotinylated anti-rabbit IgG (Vector, USA; 1∶200 for 2 h). After washing sections were incubated in Cy5-conjugated streptavidin (Jackson ImmunoResearch, USA; 1:1.500 for 2 h). To test the specificity of the staining, some sections were incubated with omission of the primary antibody. Moreover, it has been shown that this antibody (ab31895; Abcam) specifically detects TRPV1 positive neurons in rats and mice, and it further has convincingly been demonstrated that this staining was absent in TRPV1^−/−^ mice [72].

(2) For visualization of neuronal cells, sections were stained using NeuroTrace green fluorescent Nissl stain (1:300; Molecular Probes, Netherlands; for 20 min) that binds to the Nissl substance, which is present exclusively in the somata of neuronal cells.

(3) For nuclear staining, the sections were afterwards counterstained with 4,6-diamidino-2-phenylindol (DAPI, Molecular Probes, Netherlands; 1:15000), washed and coverslipped in fluorescent mounting medium (DAKO, USA).

Thus, in a section, cell nuclei could be identified by their blue fluorescence (DAPI), neurons could be identified by their green fluorescence (NeuroTrace green fluorescent Nissl stain) and TRPV1 immunoreactivity could be identified by the red signal (Cy5 labeling). A microscope (Axioplan 2 imaging; Zeiss, Germany) fitted for fluorescence and equipped with a computer-driven digital camera (Axiocam; Zeiss, Germany), a motorized z-stage and a 40× objective was used. Images of one focal plane were captured by a microscope-mounted digital camera under the control of the software Axiovision (Zeiss, Germany). Since we analyzed triple-stained material, multichannel-images were captured. A counting frame of 10.000 µm^2^ (Region of Interest; ROI) was superimposed on the calibrated images and cells were counted within this area using the image processing and analysis program ImageTool (UTHSCSA, USA) as described before [73]–[75]. The numerical densities were determined: (1) Densities of DAPI-labeled cell nuclei (readout for the cellular density) were determined by counting the numbers of DAPI-stained cell nuclei which were within a counting-frame of 10.000 µm^2^ (ROI). (2) Densities of NeuroTrace-labelled cells were determined by counting the number of NeuroTrace-stained cells within the ROI. (3) Densities of TRPV1-immunopositive cells were determined by counting the number of TRPV1-positive cells within the ROI. (4) Densities of TRPV1-immunopositive neurons were determined by counting the number of TRPV1/NeuroTrace double positive cells within the ROI. (5) Densities of non-neuronal TRPV1-immunopositive cells were determined by counting the number of TRPV1 positive cells (displaying no NeuroTrace signal) within the ROI. Since section compression and loss of particles (“lost caps”) can contribute to z-axis distortion and bias cell-counting, vibratome sections have been used, since they do not display a loss of fragments below the average density, as we have shown previously [76]. Moreover, since we have used vibratome sections, no guard zones were used [76]. Densities were calculated within the same ROI for the BL, LA and CE, which enables a direct comparison of the densities of the labeled cells in different brain nuclei, since the obtained values are not biased by the different sizes of these brain areas. Data was expressed as mean ± SEM. One-way ANOVA followed by Tukey's post-hoc test was performed using Prism 5.03 (GraphPad Software Inc., USA).

### Electrophysiology

#### Preparation and extracellular recording

Since isoflurane causes a sensitization of TRPV1 [46], mice were anesthetized with ether in the majority of recordings and decapitated. For extracellular recordings, brains were quickly removed and placed in ice-cold ACSF (in mM: 129 NaCl, 3 KCl, 1.6 CaCl_2_, 1.8 MgSO_4_, 1.25 NaH_2_PO_4_, 10 glucose, and 21 NaHCO_3_). For extracellular recordings hemisected horizontal slices derived from adult mice or coronal slices (400 µm) derived from juvenile and adult mice were prepared with a vibroslicer (Campden Instruments, Silbey, UK). The appropriate slices were then placed in an interface chamber and allowed to equilibrate for 120 min at 35°C. They were superfused continuously with ACSF (1.2 mL/min). The pH was maintained at 7.4 (95% O_2_ and 5% CO_2_). Glass microelectrodes (Science Products, Hofheim, Germany) were filled with ACSF (tip resistance 3 MΩ) and placed in the LA to record field potentials. Bipolar stimulation electrodes were used to stimulate EC fibers or fibers running through the LA (intranuclear stimulation site; IN, see also [32]).

#### Whole-cell patch clamp recordings

For patch clamp recordings standard procedures were also used to prepare 300 µM thick horizontal slices derived from juvenile mice (P18–28) (mEPSCs, mIPSCs) or coronal slices derived from adult mice for LTP experiments. Whole-cell recordings from LA projection neurons were performed under submerged conditions. Cells were visually identified with infrared videomicroscopy using an upright microscope equipped with a 40× water-immersion objective. For recordings of mEPSCs and mIPSCs at room temperature pipettes were filled with an intracellular solution containing (in mM): 130 Cs-gluconate, 8 NaCl, 2 MgCl_2_, 0.5 EGTA, 2 Na-ATP, 0.4 Na-GTP, 10 HEPES. The pH was adjusted to 7.4 with KOH. For recordings of sIPSCs Cs-gluconate was replaced by CsCl. For patch clamp recordings of LTP at 35°C Cs-gluconate was replaced by K-gluconate. Patch pipettes with a tip opening of 1–3 µm diameter were pulled from borosilicate glass capillaries (Science Product GmbH, Hofheim, Germany). For stimulation the same bipolar electrodes as used for extracellular recordings were used to stimulate EC fibers.

#### Stimulation parameters

An input/output (I/O) response curve was constructed by varying the intensity of single pulse stimulation and averaging six responses per intensity. The stimulus intensity that evoked a mean field potential equal to 50% of the maximum response was then used for all subsequent stimulations. After determination of I/O curves, single stimuli were applied for at least 30 min, and responses were monitored. Single stimuli (duration of 100 µs) were presented every 10 s. Once a stable baseline of responses was obtained for at least 20 min, we either (1) recorded I/O in the presence of capsaicin and then continued single pulse stimulation for 15 min, or (2) in drug-free conditions, directly delivered high-frequency stimulation (HFS) as two trains at 100 Hz (duration: 1s, 30 s apart). The HFS paradigm has been chosen since theta burst stimulation did not produce consistent and reliable induction of LA-LTP.

### Drug application

All drugs were bath-applied at the indicated concentrations, starting at least 30 min before HFS applied to slices located in the interface chamber. We alternated between control and treatment experiments to account for potential day-to-day and time-of-day differences. It is to consider that, in contrast to submerged conditions (Fig. 3 and 4), in the interface condition the half-time to equilibrium is about 25 min [77]. However, drug-induced changes in LTP magnitude of the amygdala were also investigated in other studies using interface chambers [24], [78], [79]. We used capsaicin as agonist and capsazepine and AMG9810 as TRPV1 antagonists. The antagonist capsazepine is only able to inhibit a capsaicin-evoked response of mouse TRPV1 with an IC_50_ of 1426 ± 316 nM [80]. Despite capsazepine exhibiting a low potency relative to all other TRPV1 antagonists, it has been a fundamental pharmacological tool in defining the effects of TRPV1 activation [38]. To block NOS activity L-NAME was applied. As a CB1 antagonist AM251 (IC_50_, 1.1 µM [81]) was used. All drugs and additionally, anandamide, SR95531, CGP55845 (GABA_B_ receptor antagonist), APV, CNQX, TTX and bicuculline were obtained from Tocris Bioscience Bristol, UK.

### Data analysis

Extracellular data was collected and averaged with the custom-made software Signal 2 (Cambridge Electronic Design, UK). We defined the field potential amplitude as the absolute DC voltage of a vertical line running from the minimal point of the field potential to its intersection with a line running tangential to the points of field potential onset and offset. It is assumed that the recorded negative wave reflects a summation of both excitatory postsynaptic potentials (EPSPs). The slope measure in the LA is more sensitive to variability and signal noise, making it more difficult to analyze [82]. We, therefore, analyzed the amplitude of field potentials in the present study.

Patch clamp data was recorded with an Axopatch 200B, filtered at 2 kHz, digitized at 10 kHz, and acquired using the Clampex 9.0 software (Axon Instruments Inc., Foster City, CA). Series resistance was monitored throughout the experiment; whereby, recordings with changes of more than 15% were discarded. The liquid junction potential was not compensated.

### Statistical analysis

Significant differences between groups were calculated by the Mann-Whitney test (Software GraphPad Prism 5) because the distribution of the data was not Gaussian. Nevertheless, the same results we could obtain with ANOVA. Significance was set to *p*<0.05. To express and compare changes of field potential amplitudes between the animal groups, we averaged responses from the 57–60 min period after HFS. Patch clamp data was analyzed with ClampFit9.0 (Axon Instruments, Union City, CA, USA). Frequency changes were calculated by averaging in each cell 3 min. Significant differences between groups were calculated by the Mann-Whitney test (Software GraphPad Prism 5). Averaged data was expressed as mean ± SEM. Kolmogorov-Smirnov test was used to determine whether the distributions of two data sets (Fig. 4) were significantly different.
